# Effects of excessive bleaching on hair: comparative analysis of external morphology and internal microstructure

**DOI:** 10.1186/s42649-024-00104-0

**Published:** 2024-12-17

**Authors:** Dong Heui Kim, Seung Hyun Oh, Byung Soo Chang

**Affiliations:** 1 Department of Convergence Medicine, Yonsei University Wonju College of Medicine, Wonju, Gangwon, 26426 Republic of Korea; 2 Department of Fashion Management, Fashion Institute of Technology SUNY Korea, Incheon, 21985 Republic of Korea; 3 Department of Cosmetology, Hanseo University, Seosan, Chungnam 31962 Republic of Korea

**Keywords:** Bleached hair, Cortex, Cuticle layer, Hair, Melanin granule

## Abstract

This study investigates the impact of excessive bleaching on the external morphology and internal microstructure of hair, compared to untreated hair. Using scanning electron microscopy (SEM) and transmission electron microscopy (TEM), we observed significant changes in both the surface and internal structures of bleached hair. SEM analysis of normal hair revealed a relatively clean surface with intact cuticle scales, while bleached hair showed brittle, torn scales with a rough appearance. In areas where the cuticle was broken, remnants of endocuticle debris were still attached, contributing to the rough surface. Complete separation of the cuticle layer resulted in numerous longitudinal fissures along the exposed cortical surface of bleached hair. TEM analysis further confirmed distinct differences; in normal hair, the cuticle layer and cortex were well-separated, and a small hole was observed within the endocuticle of the cuticle cells. Conversely, in bleached hair, the cuticle layer was separated from the cortex, with numerous pores formed by the dissolution of melanin granules scattered within the cortex, specifically between the macrofibrils. No melanin granules were detected in the cortex of bleached hair, although the macrofibril structure remained intact. The findings clearly indicate that excessive bleaching leads to the loss of the cuticle layer, exposing the cortex and significantly altering the hair’s structural integrity.

## Introduction

Modern humans engage in various hairdressing practices to express their individuality and make a positive impression on others. Hair treatments such as dyeing, perming, and bleaching are readily available in hair salons. Among these, bleaching is a popular cosmetic procedure used to lighten natural hair or prepare it for a wide range of colors. As a result, the frequent use of hair bleaching procedures in salons is leading to an increase in hair fiber damage.

Bleach is used to lighten people’s hair. The bleach reacts with the chromophores within the melanin granules in the hair cortex, breaking them down through an irreversible chemical reaction to achieve a lighter hair color. Melanin granules are decomposed by alkaline oxidizing agents of high pH level. These processes involve dissolving the disulfide bond of cysteine amino acids in the granules with an oxidizing agent (Pavani et al. 2018).

Melanin granules are found in the cortical cells of the hair cortex, which run parallel to the hair fibers and are tightly packed. These cells are composed of amorphous sulfur-containing proteins and keratin filaments. Keratin itself is colorless, so hair color is determined by the presence or absence of melanin granules in the cortex (Wilk et al. 1995; Hearle 2000).

Ammonium persulfate, a common bleaching agent, is alkaline. During the bleaching process, oxidation causes the decomposition of melanin granules and partial destruction of the disulfide bonds in keratin proteins that make up the cuticle and cortex layers of the hair, weakening the hair structure. As a result, the scales of cuticle cells on the hair surface become separated or damaged, leading to the formation of holes in the cytoplasm of both cuticle and cortical cells (Tate et al. 1993; Bolduc and Shapiro 2001).

In a single bleaching process, melanin granules are not fully decomposed, with some remnants persisting around the limited membrane of the granules. During hair bleaching, rapid decomposition and dissolution of melanin granule molecules occur over a specific time period. As the chromophore is destroyed, the color of brown hair transitions from red to yellow.

When hair is oxidized using ammonium persulfate, a strong alkaline agent, along with solvent extraction and chlorine-based ingredients, the bleaching mechanism alters the protein and lipid components of the hair. The lipid content decreases, facilitating the diffusion of the bleaching agent into the hair. Decolorization intensifies as high concentrations of hydrogen peroxide penetrate the cortex, generating more free melanin, cleaving melanin granules, and causing oxidative damage (Pan et al. 2019). While a single bleaching process is less effective, repeated bleaching significantly reduces endogenous lipids and alters amino acids in cuticle cells (Masukawa et al. 2004). Consequently, both physical and photochemical damage to the hair fiber occur due to changes in its protein and lipid components.

Hair damage and weathering are caused by chemical stimuli, such as bleaching agents, hair dyes, and perming solutions, as well as physical stimuli from the external environment (Chang et al. 2005; Lee and Chang 2019). Repeated hair treatments like perming, dyeing, and bleaching weaken hair fibers over time. Damage to the cuticle layer exposes the cortex to environmental factors, leading to the eventual cleavage and destruction of hair fibers when exposed macrofibrils are in constant contact with moisture. Macrofibrils are the main structural component of the hair cortex.

Hair damage from weathering and repeated bleaching, particularly discoloration, is primarily due to the loss of 18-methyleicosanoic acid in the hair’s epicuticle (Masukawa et al. 2004). While various studies have focused on the physicochemical and morphological properties of hair (Kon et al. 1998; Richena et al. 2014; Smith et al. 2017; Lee and Chang 2019), there is a lack of research on the microstructural damage to the cortex and the decomposition of melanin granules.

In this study, we used scanning electron microscopy to observe surface damage to the cuticle layer from excessive bleaching, and high-magnification transmission electron microscopy to examine microstructural changes in the cortex.

## Materials and methods

### Experimental materials

In this study, hair from a female in her twenties with healthy, untreated hair (no chemical treatments such as dyeing, bleaching, or perming) was used as the normal hair sample. For the bleaching process, a powder-type alkaline agent (pH 9–11) was used in combination with a 6% hydrogen peroxide (H₂O₂) oxidizing agent from Blondor, Wella Korea. The bleaching process involved mixing the alkaline and oxidizing agents in a 1:1 ratio. The mixture was applied starting approximately 1 cm away from the hair root. To accelerate the bleaching reaction, heat was applied using a hair dryer from approximately 20 cm for 1-2 min, and the hair was left for about 20 min before thoroughly rinsing with running water and allowing it to air dry. This bleaching process was repeated three times, after which the bleached hair was collected for testing. To observe the external morphology and internal microstructure of both bleached and normal hair using an electron microscope, each sample was cut to approximately 1 mm in length using a sharp double-edged razor.

### Scanning electron microscopy

Samples of both bleached and normal hair were pre-fixed in 2.5% paraformaldehyde-glutaraldehyde at 4℃ in a phosphate buffer (pH 7.4). After two washes with phosphate buffer solution, the samples were post-fixed in 1% osmium tetroxide (OsO₄) in the same buffer at 4℃ for 1 h. Following fixation, the samples were washed again in the phosphate buffer, dehydrated using a graded ethanol series, and replaced with isoamyl acetate. The treated hair samples were then dried using a critical point dryer (SCP-II, Hitachi, Japan). Once dried, the hair was coated with a 20 nm layer of platinum using an ion coater (IB-5, Eiko, Japan), and observed under a scanning electron microscope (S-4700, Hitachi, Japan) at 15 kV.

### Transmission electron microscopy

For the observation of the internal microstructure of normal and bleached hair, each sample, cut to approximately 3 mm in length, was placed in a 5 ml penicillin glass bottle and pre-fixed in 2.5% paraformaldehyde-glutaraldehyde (4℃, phosphate buffer, pH 7.4) for 1 h. The samples were then washed twice with phosphate buffer solution (4℃, 0.4 M phosphate buffer, pH 7.4) for 15 min each. Post-fixation was performed with 1% OsO₄ (4℃, 0.4 M phosphate buffer, pH 7.4) for 1 h.

After fixation, each sample was washed twice in the same buffer solution and dehydrated through a graded ethanol series. Following dehydration, the samples were treated with propylene oxide and embedded in an Epon-Araldite mixture. Polymerization was carried out in a vacuum drying oven (DPF-31, Yamaro, Japan) at 60℃ for 36 h. The embedded blocks were sectioned into 1 μm semi-thin slices using an ultramicrotome (LKB-2088, Olympus, Japan) and stained with toluidine blue for optical microscope observation.

For further microstructural analysis, ultra-thin sections were prepared and mounted onto copper grids, then double-stained with uranyl acetate and lead citrate. These sections were observed using a transmission electron microscope (H-7500, Hitachi, Japan) at 100 kV.

## Results

**Fig. 1:**
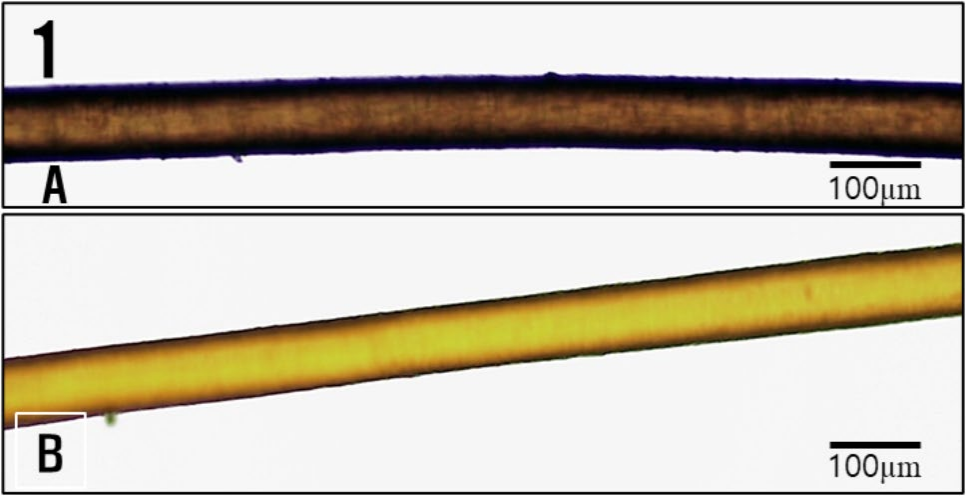
Optical micrographs of normal hair and bleached hair. (**A**): normal hair, (**B**): bleached hair

In this study, optical microscope observations revealed that the natural color of normal hair was brown (Fig. 1A), whereas bleached hair appeared bright yellow (Fig. 1B).

**Fig. 2:**
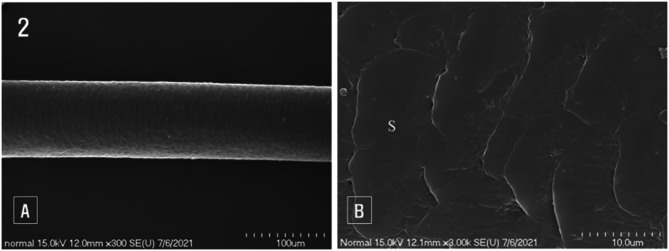
Scanning electron microscopic images of normal hair. (**A**): Low magnification image showing the clear surface of the hair, (**B**): High magnification image highlighting the scales (S) covering the hair surface

Low-magnification scanning electron microscopy of normal hair revealed no significant surface damage (Fig. 2A). At high magnification, the tips of the exposed cuticle scales appeared gently rounded. However, the scales exhibited damage, with some partially separated and their edges slightly broken and irregular (Fig. 2B).

**Fig. 3:**
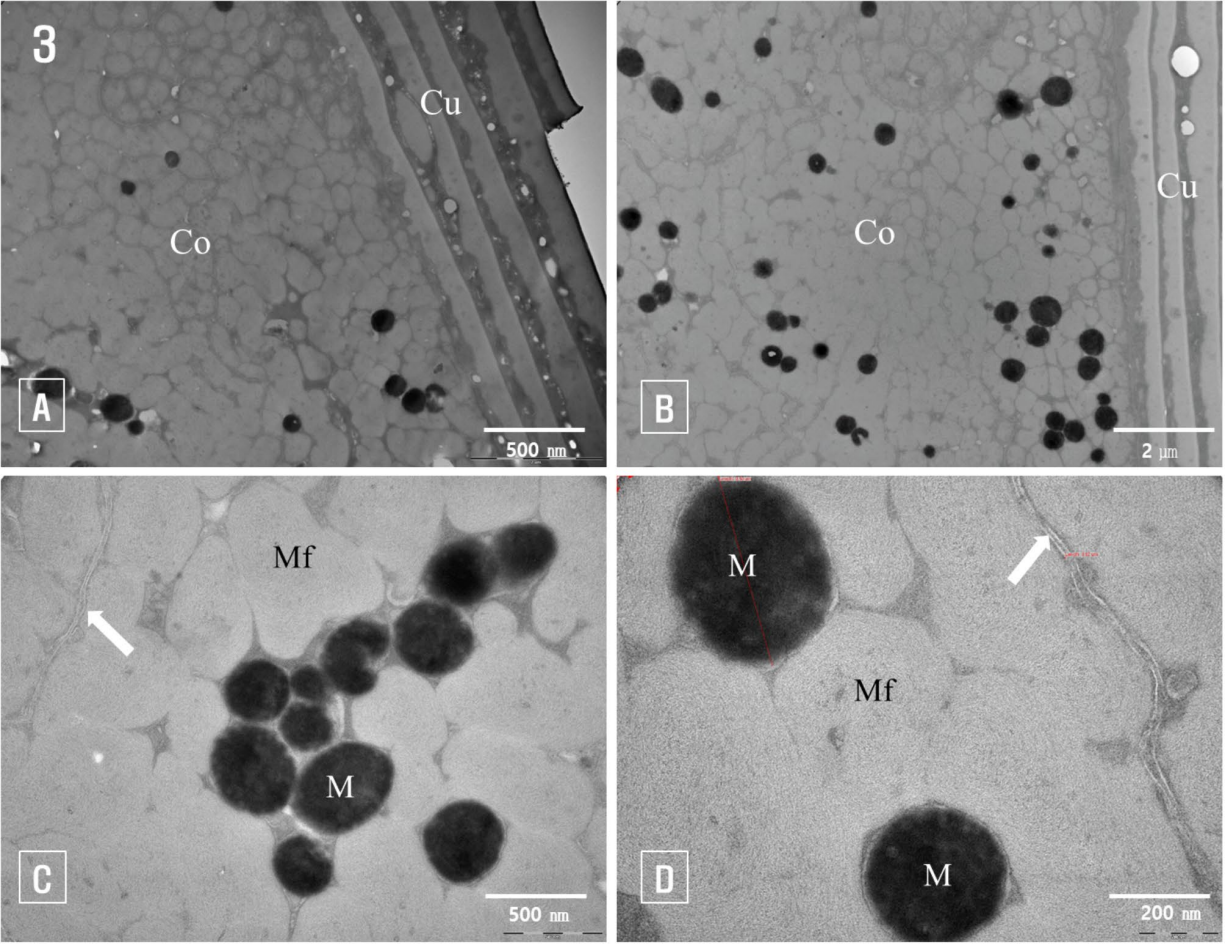
Transmission electron micrographs of normal hair fibers. (**A**): The cuticle layer (Cu) is composed of several layers of cuticle cells. Endocuticles with high electron density have many pores, (**B**): Cortex (Co) and cuticle layer (Cu) of normal hair, (**C**): Melanin granules (M) present between macrofibrils in the cortex, (**D**): Macrofibrils (Mf) and melanin granules (M) surrounded by cortical cells membrane complex (arrow)

Under transmission electron microscopy, the cuticle layer of normal hair was observed to be surrounded by several layers of cuticle cells. Each cell was distinctly divided into an exocuticle and an endocuticle based on differences in electron density, with the endocuticle exhibiting higher electron density than the exocuticle (Fig. 3A). The endocuticle is more susceptible to damage, showing numerous holes with diameters ranging from 0.1 μm to 0.5 μm (Fig. 3A).

In contrast, the exocuticle had relatively lower electron density and showed no evidence of holes or damage (Fig. 3B).

Cortical cells in the cortex were tightly packed and exhibited holes similar to those in the endocuticle, observed around melanin granules, between macrofibrils, and among cell remnants (Fig. 3A, B).

In transverse sections of the hair fiber, cortical cells appeared polygonal and irregular, with cytoplasm filled with macrofibrils and melanin granules. Destroyed nuclei and organelles displayed high electron density and irregular shapes (Fig. 3A, C). The cross-sections of macrofibrils were elliptical, with diameters ranging from approximately 0.5 μm to 1 μm. The microfibrils within the macrofibrils were arranged in a fingerprint-like pattern. Membrane complexes between cortical cells had a thickness of approximately 20 nm (Fig. 3C, D).

Melanin granules between the macrofibrils were aligned along the longitudinal axis of the hair fibers, resulting in circular or oval cross-sections. The size of the melanin granules varied depending on the cut site, with a measured diameter of approximately 0.5 μm in the transverse section (Fig. 3D).

**Fig. 4:**
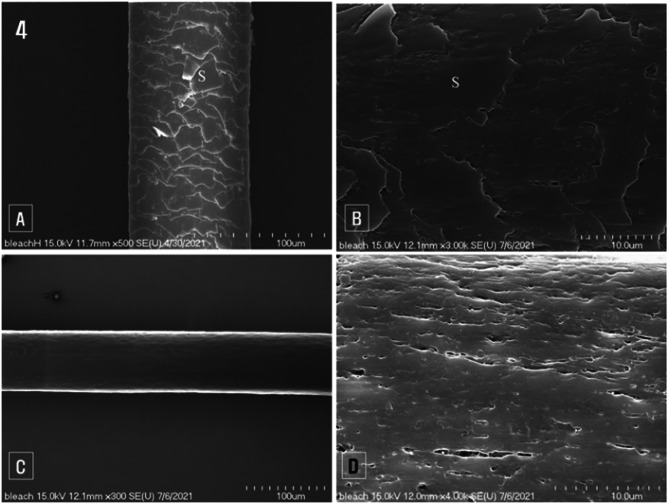
Scanning electron micrographs of bleached hair. (**A**): Low magnification image of bleached hair, (**B**): Scales (S) observed to be broken or separated on the hair surface, (**C**): Complete removal of the cuticle layer leaving the cortex exposed, (**D**): Fissures formed along the long axis of the hair fiber on the surface of the exposed cortex

Under scanning electron microscopy, the surface of bleached hair appeared rough, with broken and detached scales (Fig. 4A). At high magnification, the outermost cuticle scales were observed to be separated and peeled off, revealing the underlying surface of the cuticle cells. Debris from the endocuticle adhered to the area where scales had fallen off, giving the surface a messy appearance (Fig. 4B). The rough texture of the bleached hair was due to the separation of cuticle cells and the attachment of endocuticle fragments to the surface of the underlying cuticle cells (Fig. 4B).

In cases of severe damage, the cuticle layer was completely stripped away, exposing the cortex underneath (Fig. 4C). High magnification revealed that, in these severely damaged hairs, the cuticle layer was entirely removed, exposing the cortex with noticeable longitudinal cracks along the hair fibers (Fig. 4D).

**Fig. 5:**
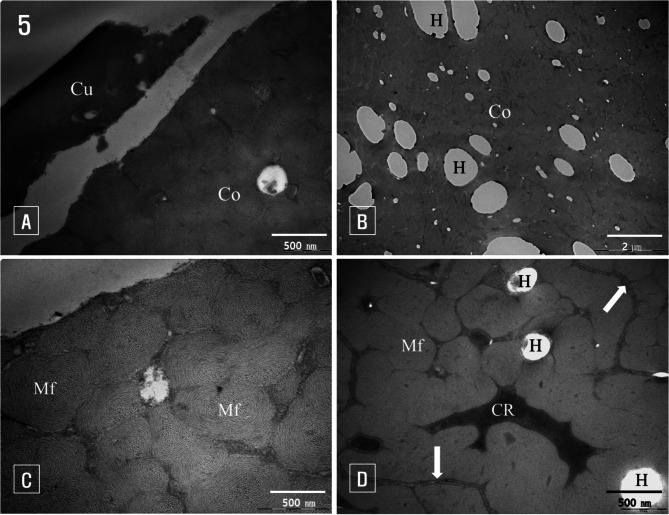
Transmission electron micrographs of bleached hair. (**A**): Cuticle cells (Cu) separated from the cortex (Co), (**B**): Holes (H) of various sizes visible in the cortex (Co), with no melanin granules present, (**C**): Outer cortex where the cuticle layer is completely removed, showing intact macrofibrils (Mf), (**D**): Macrofibrils (Mf) and cortical cell remnants (CR) within the cortex, with visible holes (H) and membrane complexes (arrows)

Under transmission electron microscopy, bleached hair revealed that the cuticle layer was largely removed, exposing the cortex. The cuticle cells still attached to the hair fibers were separated from the cortex. Numerous holes, formed by the dissolution of melanin granules within cortical cells, were observed. These holes appeared as large pores due to physical deformation (Fig. 5A).

Complete melanin granules were not observed in the cortex of bleached hair. Following treatment with the bleaching agent, melanin granules dissolved, although some undissolved fragments remained at the edges of the holes (Fig. 5B). As shown in Fig. 5C, the macrofibrils within the cortical cells retained their shape. In transverse sections, the microfibrils were arranged in a fingerprint-like pattern, with a matrix filling the spaces between them (Fig. 5C). Additionally, cell remnants from lysed organelles and nuclei were clearly visible between the macrofibrils, distinguished by their higher electron density (Fig. 5D).

## Discussion

Modern humans bleach their hair to achieve various shades by removing the melanin granules in the hair cortex. Typically, bleaching agents are prepared by mixing an alkaline compound, such as potassium persulfate or ammonium persulfate, with up to 12% hydrogen peroxide.

During the bleaching process, the cuticle layer is the first part of the hair to come into contact with the bleach. The cuticle is composed of keratinized, anucleated squamous cells that overlap to form the outer texture of the hair (Kreplak et al. 2001; Seshadri and Bhushan 2008).

In this study, the scales on the surface of normal hair were intact, surrounding the hair fibers. However, the scales on the surface of bleached hair were damaged and broken. The function of these scales is crucial in reducing elasticity, softness, volume, gloss, ease of combing, and static electricity of the fibers, as they provide adhesion and control movement (Velasco et al. 2009; He et al. 2021). The scales are part of the cuticle cells exposed on the surface, which form the outer layers of the cuticle surrounding the cortex.

In the bleaching process, oxidation breaks some of the disulfide bonds in the keratin protein, weakening the hair’s structure and damaging the cuticle cells, leading to the formation of numerous holes in the cytoplasm (Bolduc and Shapiro 2001).

In this study, damage to the bleached hair was observed as the endocuticle, containing holes in the cuticle cells, was broken. As a result, the cuticle layer, composed of damaged cuticle cells, completely detached and peeled off, exposing the cortex. Chang and Lee (2006) noted that bleaching alters the chemical structure of the hair fiber, making it more prone to breakage and more sensitive to humidity.

Kuzuhara (2013) reported structural changes in the cuticle layer of bleached black human hair, with a decrease in electron density in the endocuticle and an increase in the exocuticle. However, in this study, no change in electron density was observed in either the endocuticle or exocuticle. Nonetheless, more holes were found in the endocuticle of bleached hair compared to normal hair. The cortex of hair fibers is filled with cortical cells, which consist of macrofibrils, melanin granules, and cell remnants. The spindle-shaped macrofibrils in the cortical cells are composed of microfibrils and matrix, each with distinct structural characteristics and amino acid composition.

Microscope revealed that the microfibrils within the transversely cut macrofibrils were arranged in regular intervals, resembling fingerprints. An amorphous matrix filled the spaces between the microfibrils, providing strength and flexibility to the keratin protein of hair (Nishikawa et al. 1998).

Microfibrils are crystalline fibrous proteins composed primarily of α-helical proteins with a low cystine disulfide bond content (Feughelman 2002). In the cortex, keratin fibers are cross-linked by disulfide bonds, which contribute to the structural stability and physical and mechanical properties of hair (Kuzuhara et al. 2007).

This study confirmed that the cortex of normal hair was keratinized, with some ultramicroscopic holes forming between the macrofibrils. In bleached hair, the cortex exhibited a porous structure, with scattered holes of varying sizes formed by the dissolution of melanin granules between the macrofibrils. The porous nature of the cortex facilitates hair weathering by absorbing moisture from the external environment.

While bleaching is widely used to lighten hair color, it causes significant damage. In this study, the surface of the bleached hair showed separation and breakage of cuticle cells. In more severe cases, the entire cuticle layer was peeled off, exposing the cortex. The exposed cortex developed long fissures along the longitudinal axis of the hair. Hair damaged in this manner absorbs moisture through cracks in the cortex, either from washing or the surrounding environment. Contact between macrofibrils in the cortex and water leads to further degradation. Such damaged hair has a visibly rough appearance.

In addition, the fibrous proteins in bleached hair are weakened due to increased cortical porosity caused by the decomposition of melanin granules and damage to the cuticle layer (Wolfram et al. 1970; Kuzuhara et al. 2007; Richena et al. 2014). Hair fibers are composed of 65–95% protein and up to 32% water, with the remainder consisting of lipids and other components. The primary protein in hair is α-keratin, and tensile strength is mainly generated in the cortex (Yang et al. 2014).

In this study, numerous holes formed in the cortex of bleached hair fibers, with larger holes resulting from continuous physicochemical stimulation. Excessive bleaching, therefore, leads to a loss of elasticity and tensile strength in the hair fibers, eventually causing breakage or splitting.

## Conclusion

As a result, excessive bleaching significantly compromises the structural integrity of hair by stripping away the cuticle layer and causing extensive internal damage. These findings underscore the importance of carefully managing hair treatments to minimize damage and maintain hair health.

An examination of the external and internal microstructural characteristics of normal and bleached hair revealed significant morphological changes in bleached hair, particularly in the cuticle layer and cortex. In contrast, normal hair showed no substantial scale separation or severe damage, though minor breakage at the tips of some scales was observed—a natural result of regular washing or friction between hair strands during daily activities.

In bleached hair, the cuticle layer exhibited significant damage, with scales separating and some cuticle cells fragmenting and peeling off, exposing the cytoplasm. Debris from the endocuticle remained attached to the hair surface as scales detached, resulting in sharp, irregular edges. In cases where the cuticle layer was fully peeled away, the underlying cortex was exposed, revealing numerous cracks. These cracks allowed external moisture to penetrate, accelerating the weathering process. Over time, the separation of macrofibrils within the hair fibers progressed, often leading to the need for trimming the damaged hair.

In bleached hair, numerous holes were observed in the endocuticle and cortex. Notably, the dissolution of melanin granules within the cortex created voids that significantly weakened the structural integrity of the hair fibers, making them more prone to breakage.

## Data Availability

Data and materials are available on request.

## Abbreviations

EDS: Energy-dispersive X-ray spectroscopy
SEM: Scanning electron microscopy
